# Supplementary material for: Preparation and Application of Fluorine-Free Finishing Agent with Excellent Water Repellency for Cotton Fabric
Source: Polymers (Basel). 2021 Sep 2;13(17):2980. doi: 10.3390/polym13172980 (PMC8433999; doi:10.3390/polym13172980)
Supplement: Supplementary file 1 [file polymers-13-02980-s001.zip › polymers-1344908-supplementary.pdf]

Supplementary Materials

Supplementary figure captions:

Figure S1. The influence of dosage of APDES on ammonia value of CAHPS.

Figure S2. The effect of dosage of HDTMS on WCA of cotton fabric.

Figure S3. The particle size distribution of silica and GPTMS-SiO_2.

Figure S4. The effect of 20 nm (a), 100 nm (b) and 1 μm (c) silica on WCA of cotton fabric.

Figure S5. FT-IR spectra of CAHPS, GPTMS-SiO_2 and rSiO_2-CAHPS.

Figure S6. TGA curves of CAHPS and rSiO_2-CAHPS.

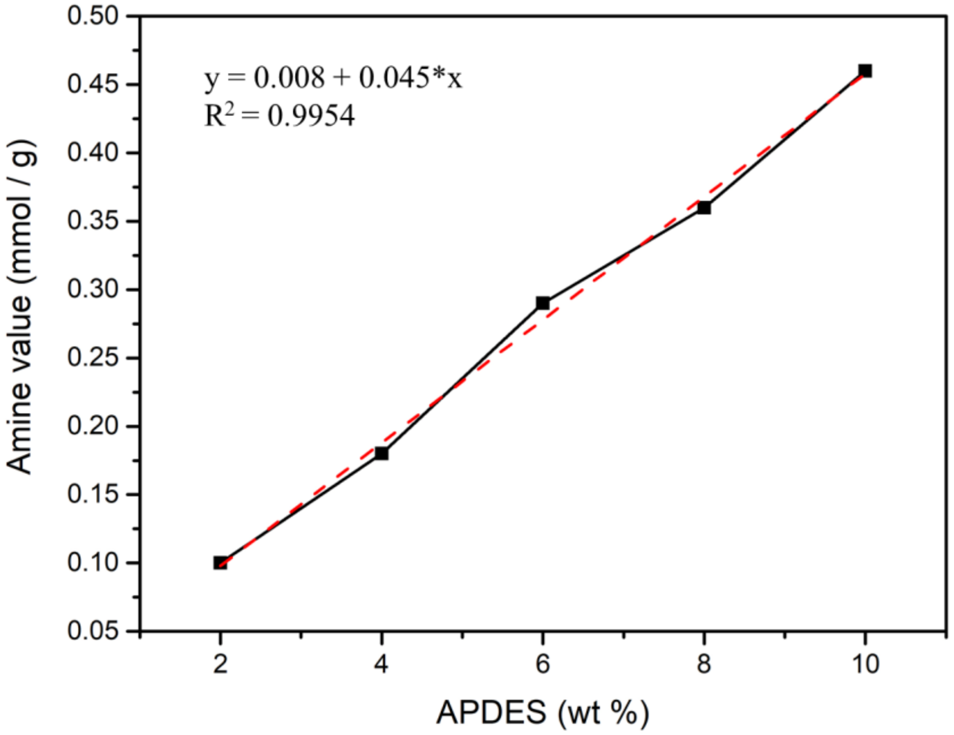

Figure S1. The influence of dosage of APDES on ammonia value of CAHPS.

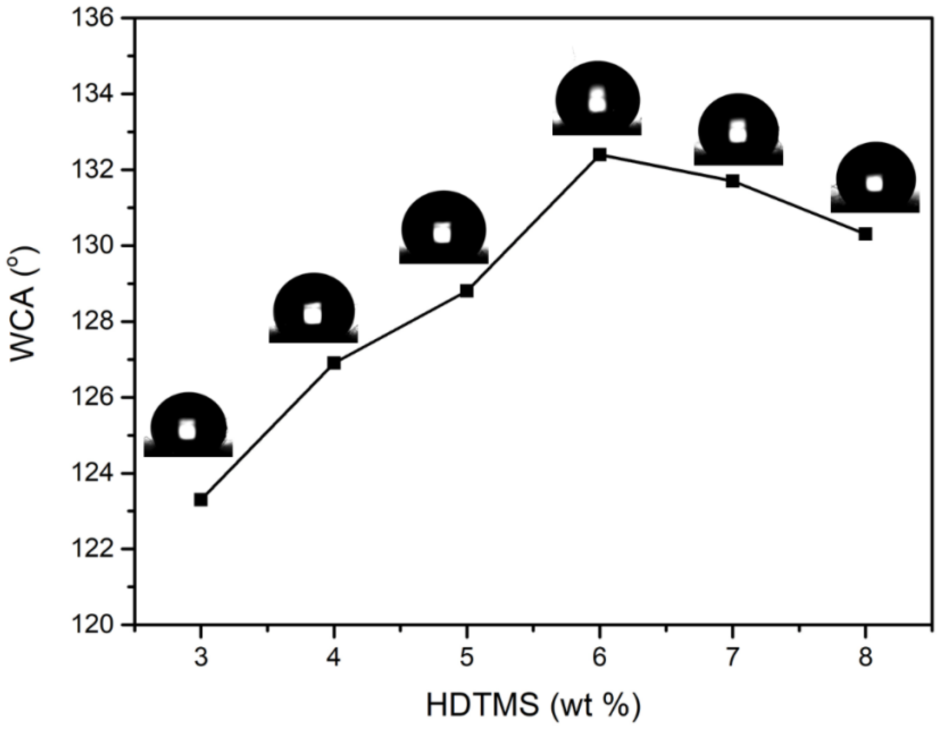

Figure S2. The effect of dosage of HDTMS on WCA of cotton fabric.

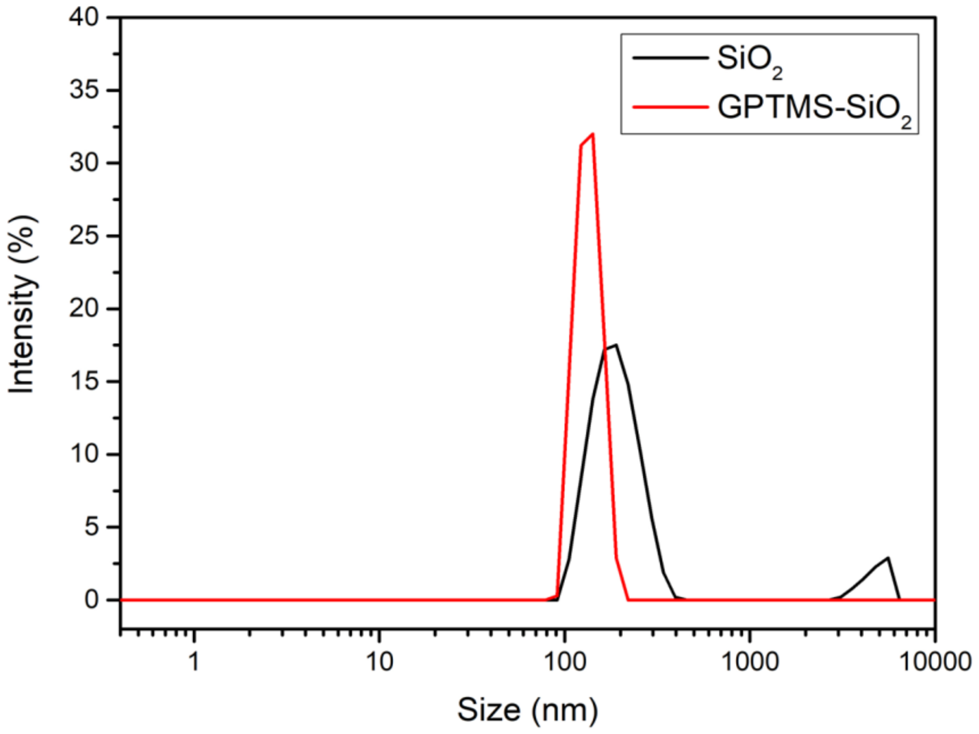

Figure S3. The particle size distribution of silica and GPTMS-SiO_2.

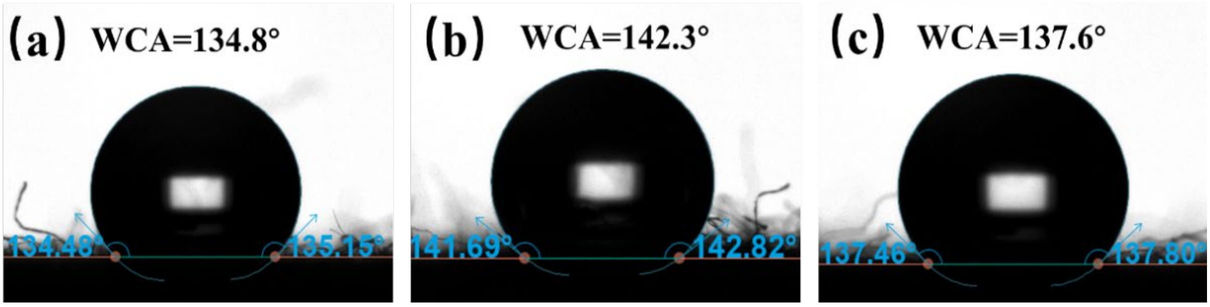

Figure S4. The effect of 20 nm (a), 100 nm (b) and 1 μm (c) silica on WCA of cotton fabric.

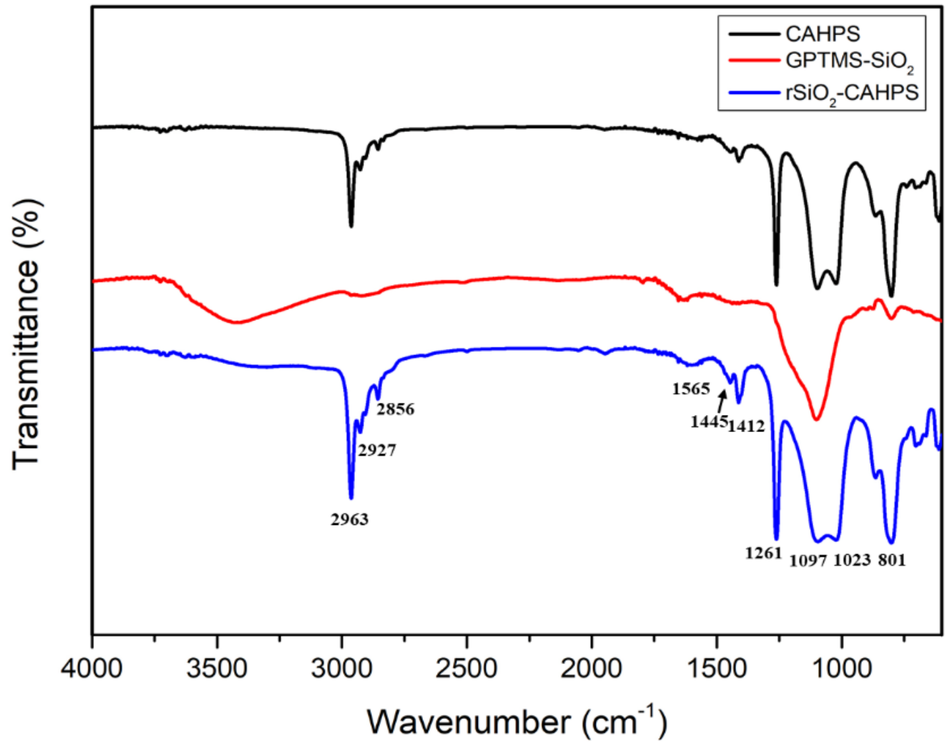

Figure S5. FT-IR spectra of CAHPS, GPTMS-SiO_2 and rSiO_2-CAHPS.

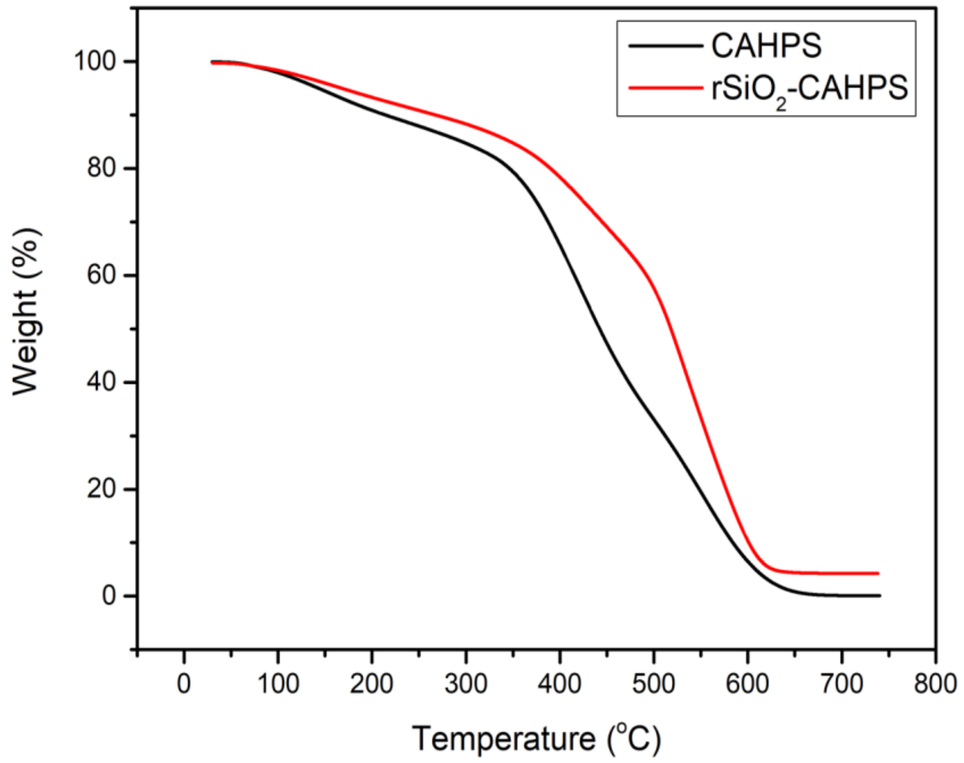

FigureS6. TGA curves of CAHPS and rSiO_2-CAHPS.
